# Supplementary figures and images for: Limitations of alignment-free tools in total RNA-seq quantification
Source: BMC Genomics. 2018 Jul 3;19:510. doi: 10.1186/s12864-018-4869-5 (PMC6042521; doi:10.1186/s12864-018-4869-5)

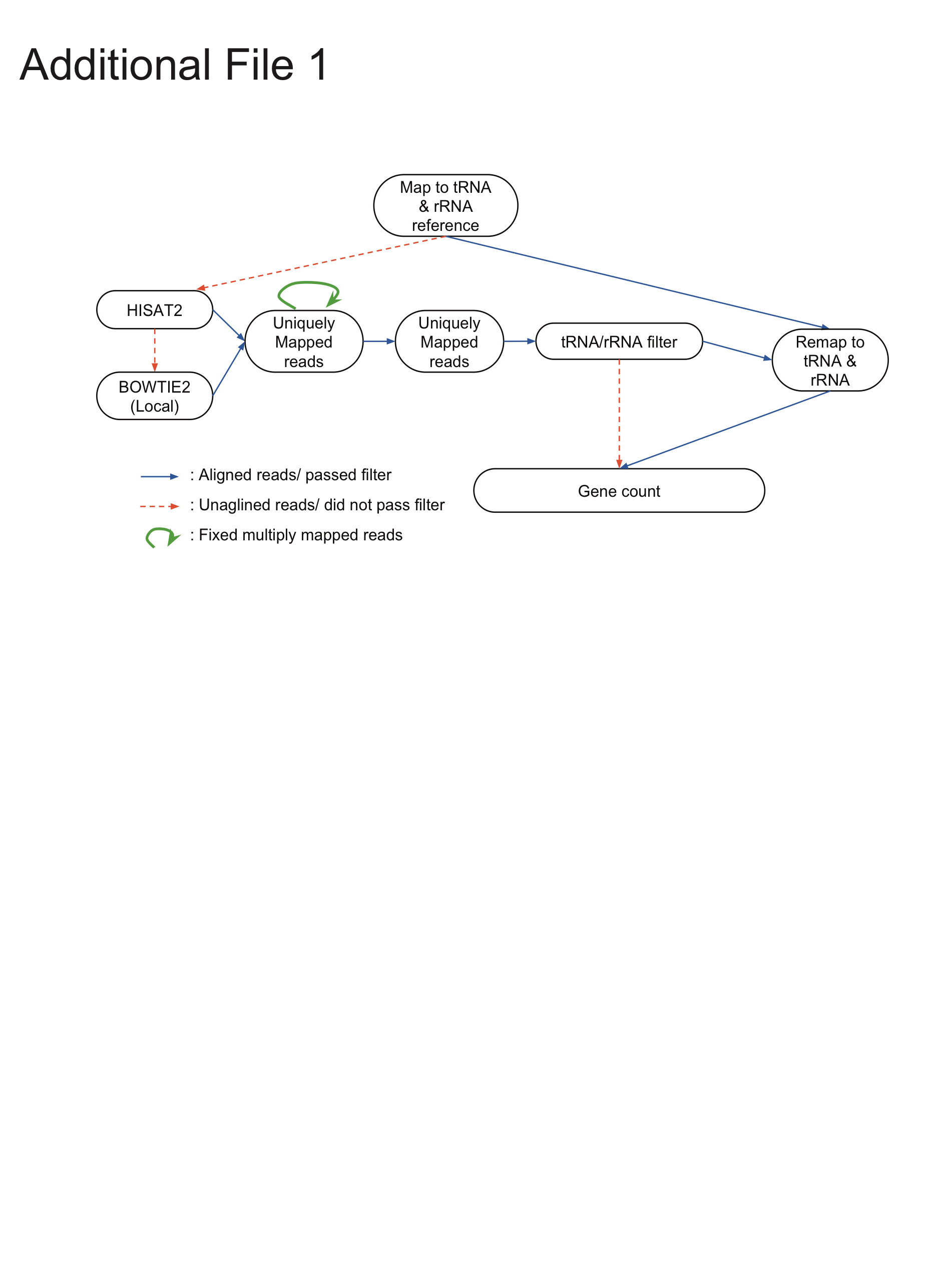

Supplement: Supplementary file 1 — Flow chart of the custom pipeline TGIRT-map. As TGIRT-seq simultaneously recovers a large fraction of small structural RNAs in addition to regular long RNAs [15, 17], TGIRT-map was designed to optimize the quantification of these small genes. The pipeline is similar to the Genobee-exceRpt small RNA-seq pipeline [34], where reads are first aligned against the tRNA and rRNA sequences to avoid ambiguous assignments in later steps. Unaligned reads (red arrow) are iteratively aligned to the human genome by HISAT2 [9] and BOWTIE2 [20] to minimize unassigned reads. Multiply-mapped reads are extracted and assigned as uniquely-mapped at loci selected by the following criteria in order: the pair of alignments (A) has the smallest insert size, (B) is mapped to ribosomal gene loci, and (C) is mapped to chromosome 1-23, X or Y, otherwise a random pair of alignments is selected. From these genome alignments, reads that aligned to tRNA or rRNA loci are extracted and combined with tRNA and rRNA reads from the first step. Finally, these combined reads are realigned to the tRNA and rRNA sequences to generate gene counts. (PNG 247 kb) [file 12864_2018_4869_MOESM1_ESM.png]

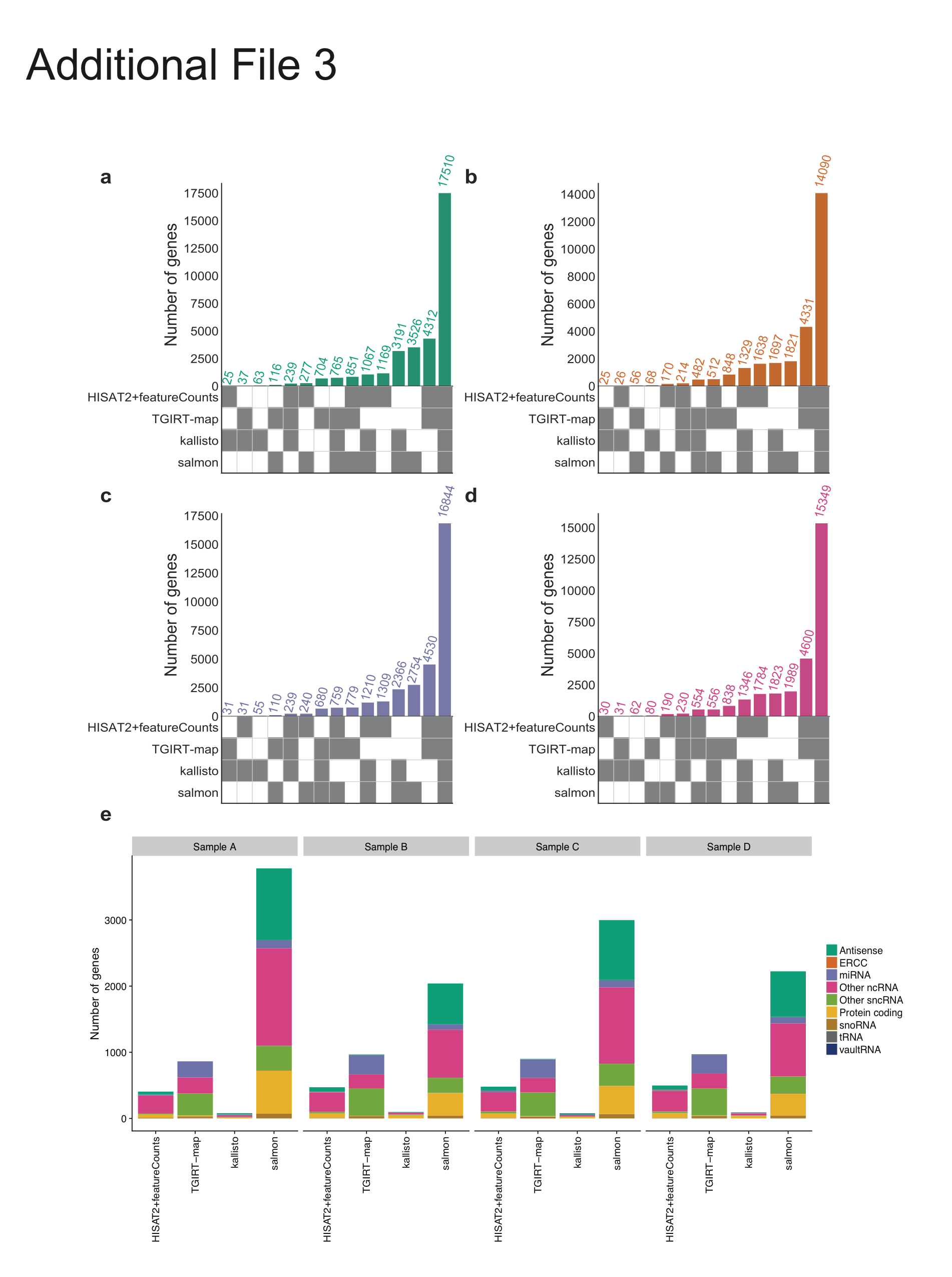

Supplement: Supplementary file 3 — Intersections of detected genes among tested pipelines. Genes with TPM > 0.1 were defined as being detected. (a–d) UpSet graphs of intersections of detected genes among pipelines. An UpSet graph describes intersections of detected genes among subsets of tested pipelines for samples A–D. Each UpSet graph contains a bar chart and a sample-comparison matrix. Bars indicate the numbers of detected genes in the specific intersection specified in the sample-comparison matrix. Each bar indicates the number of genes detected only in the intersection of the set of compared pipelines. The sample-comparison matrix at the bottom describes the set of pipelines within which the comparison is made. Grey boxes in the matrix annotate the pipelines included in the comparison. (e) Uniquely-detected genes. The heights of the stacked bars indicate the numbers of genes uniquely detected by the corresponding pipeline. Stacked bars are color-coded with gene types. (PNG 428 kb) [file 12864_2018_4869_MOESM3_ESM.png]

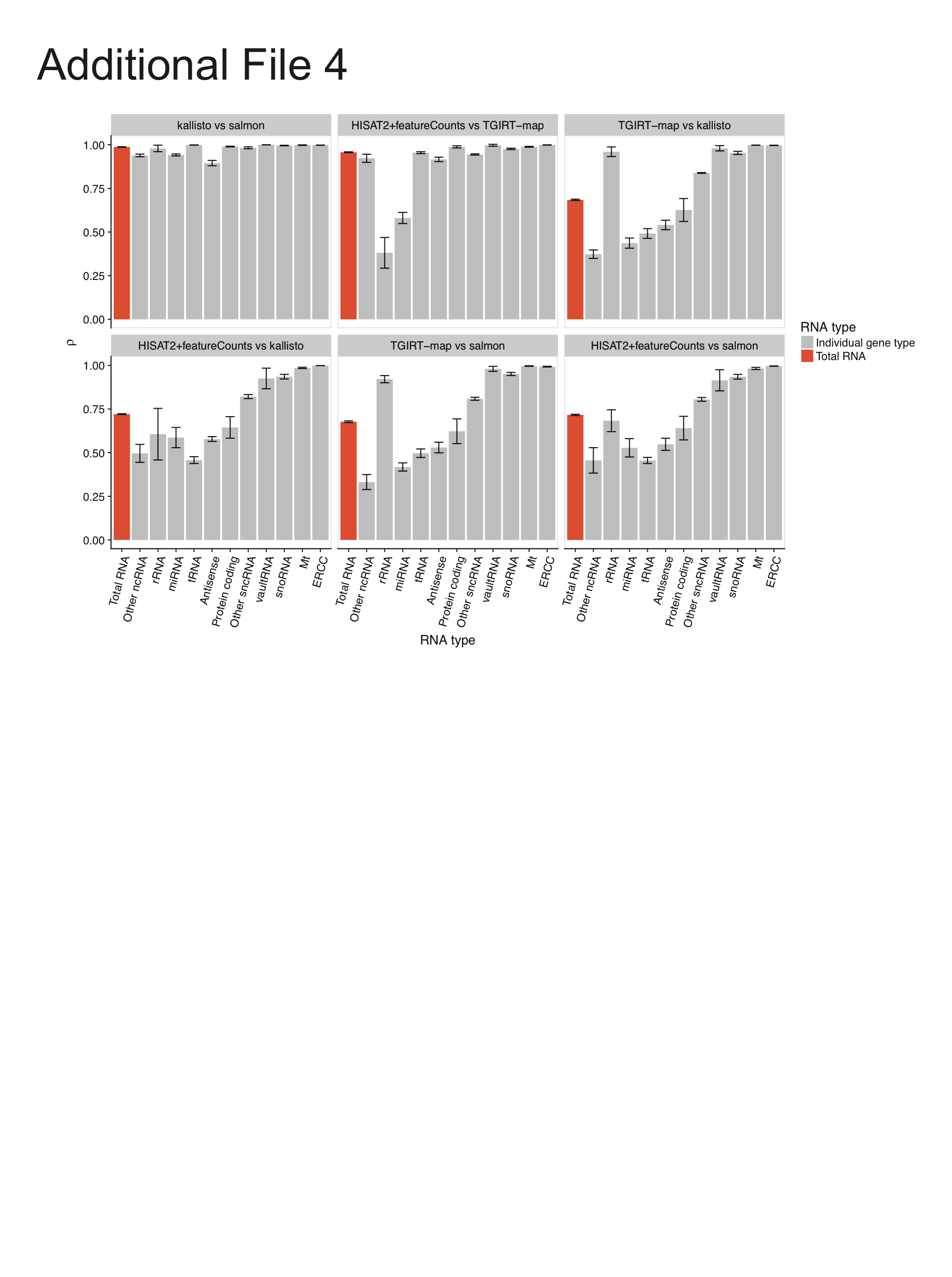

Supplement: Supplementary file 4 — Pearson’s correlation coefficients for pairwise pipeline comparisons in gene quantifications. For every pairwise pipeline comparison of each sample (A–D), Pearson’s correlation coefficients were computed using the average log2 TPM of each gene across the triplicates for each sample. Bar heights indicate average correlations among the four samples A–D. Error bars represent standard deviations among the four samples A–D. Red bars represent total RNA correlation coefficients (all genes). Grey bars indicate correlation coefficients grouped by gene type. Each panel represents a single pairwise pipeline comparison. (PNG 207 kb) [file 12864_2018_4869_MOESM4_ESM.png]

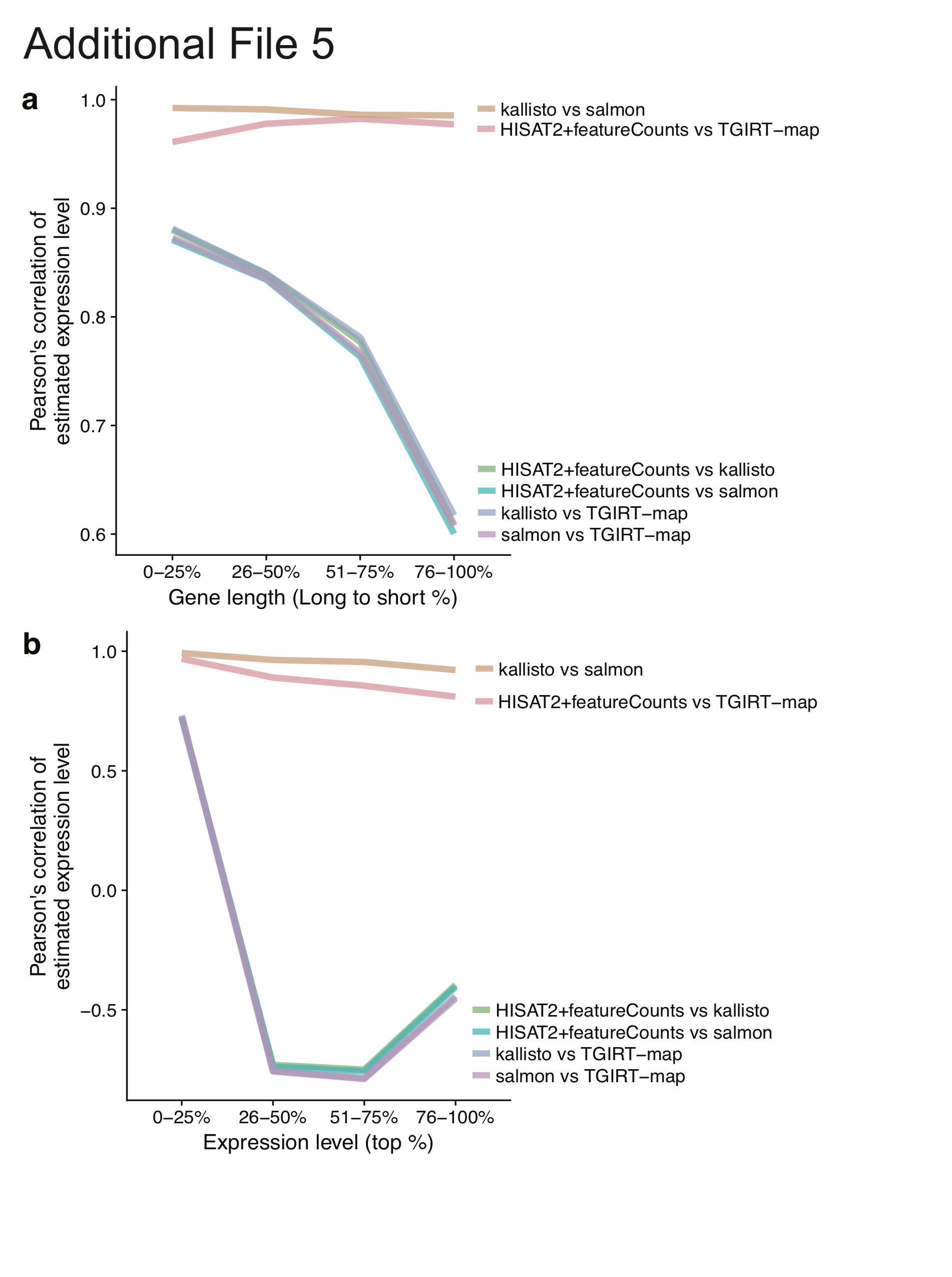

Supplement: Supplementary file 5 — Impact of gene lengths and expression levels on gene quantification correlations among pipelines. (a) Gene lengths influence gene expression estimation correlations. Genes were separated into quantile groups according to their lengths. For every quantile group from each sample, Pearson’s correlation coefficients of average TPMs for each gene across replicates were computed for every pairwise pipeline comparison and then averaged across the four samples. Plotted are the average correlations versus the quantile groups. Each line represents a pairwise comparison and is color-coded accordingly. (b) Gene expression levels influence gene expression estimate correlations. Genes were separated into quantile groups according to their average expression values (TPMs). For every quantile group from each sample, Pearson’s correlations were computed in the same way as in panel (a). Each line represents a pairwise comparison and is color-coded accordingly. (PNG 412 kb) [file 12864_2018_4869_MOESM5_ESM.png]

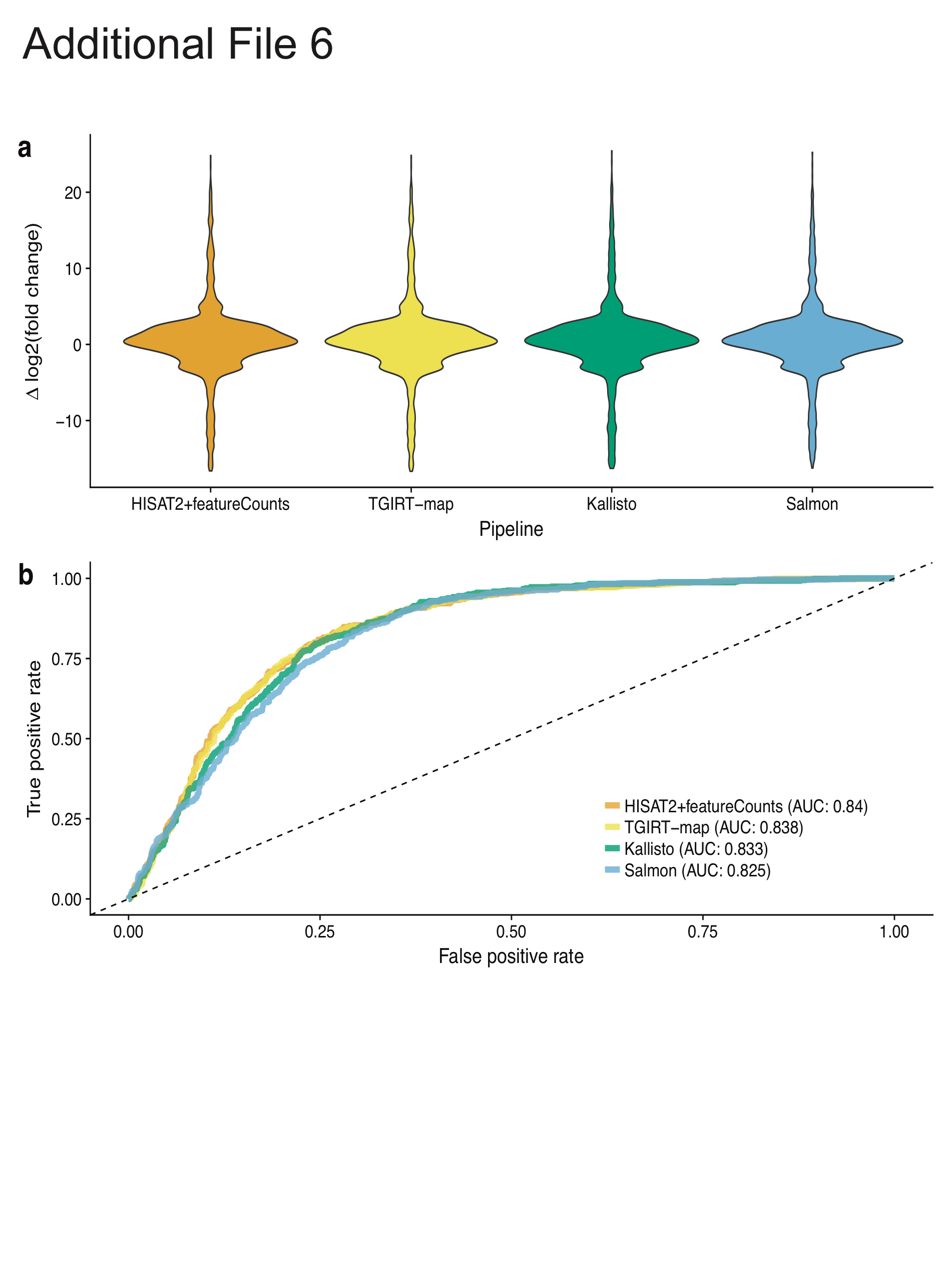

Supplement: Supplementary file 6 — Differential expression analysis of protein-coding genes. (a) Comparison of measured fold-changes between samples A and B from the TGIRT-seq analysis and the MAQC TaqMan assay. MAQC TaqMan assay data for the same samples were downloaded from the NCBI Gene Expression Omnibus (GEO), accession number GSE5350 [18]. 972 unique protein-coding genes were compared. Log2 fold-changes were calculated from MAQC data and compared to the DESeq2 results for each pipeline. Distributions of the deviations between RNA-seq and MAQC data log2 fold-changes were plotted as violin-plots. Distributions are color-coded by pipelines. (b) ROC curves for calling differentially-expressed (DE) protein-coding genes. MAQC TaqMan assay genes were binary-labeled as DE if absolute log2 fold-change > 0.5 and not DE otherwise (739 DE and 225 non-DE), as suggested by a previous study [10]. ROC curves for DESeq2 results from abundance estimates computed by each pipeline were plotted. AUCs were computed for each ROC curve to quantify performances of the differential expression caller (DESeq2) [21] on abundance estimates from each pipeline. ROC curves are color-coded by pipelines. The dotted diagonal line indicates random guessing (AUC =0.5). (PNG 352 kb) [file 12864_2018_4869_MOESM6_ESM.png]

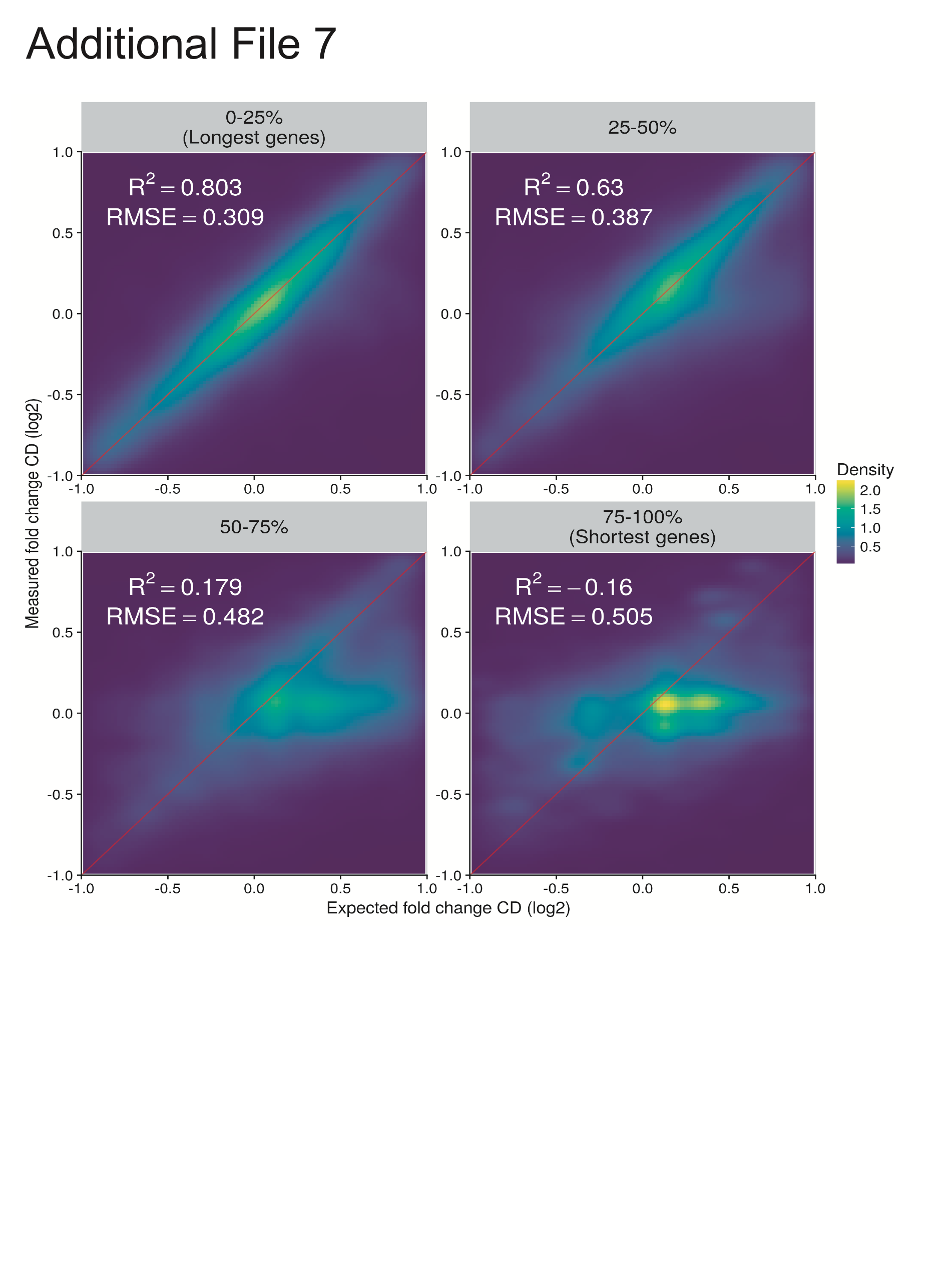

Supplement: Supplementary file 7 — Measured versus expected log2 fold-changes between samples C and D for Kallisto. Expected log2 fold-changes for each gene (denoted by red line) were constructed from (A) the Kallisto-measured log2 fold-changes between samples A and B, and (B) the known sample mix ratio of samples A and B in samples C and D, using a previously-described algorithm [19], see method. Each panel shows a two-dimensional kernel density estimation of the distribution of measured vs expected log2 fold-changes between samples C and D for genes grouped by quantile groups of gene lengths. Coefficients of determination for each group of genes are annotated as R2. Perfectly correlated log2 fold-changes are annotated by the diagonal red lines. Panels with higher R2 generally show denser trends along the red line, indicating the log2 fold-changes between samples C and D were accurately predicted by the software that recapitulated the sample-mixing ratio and thus, fitted better to the model (red line). For groups with lower R2, a denser (lighter color) horizontal trend was observed on the right side of the red line, indicating the software either over-estimated log2 fold-changes between samples A and B that led to construction of an incorrect model or under-estimated log2 fold-changes between samples C and D that led to a poor fit to the model. (PNG 471 kb) [file 12864_2018_4869_MOESM7_ESM.png]

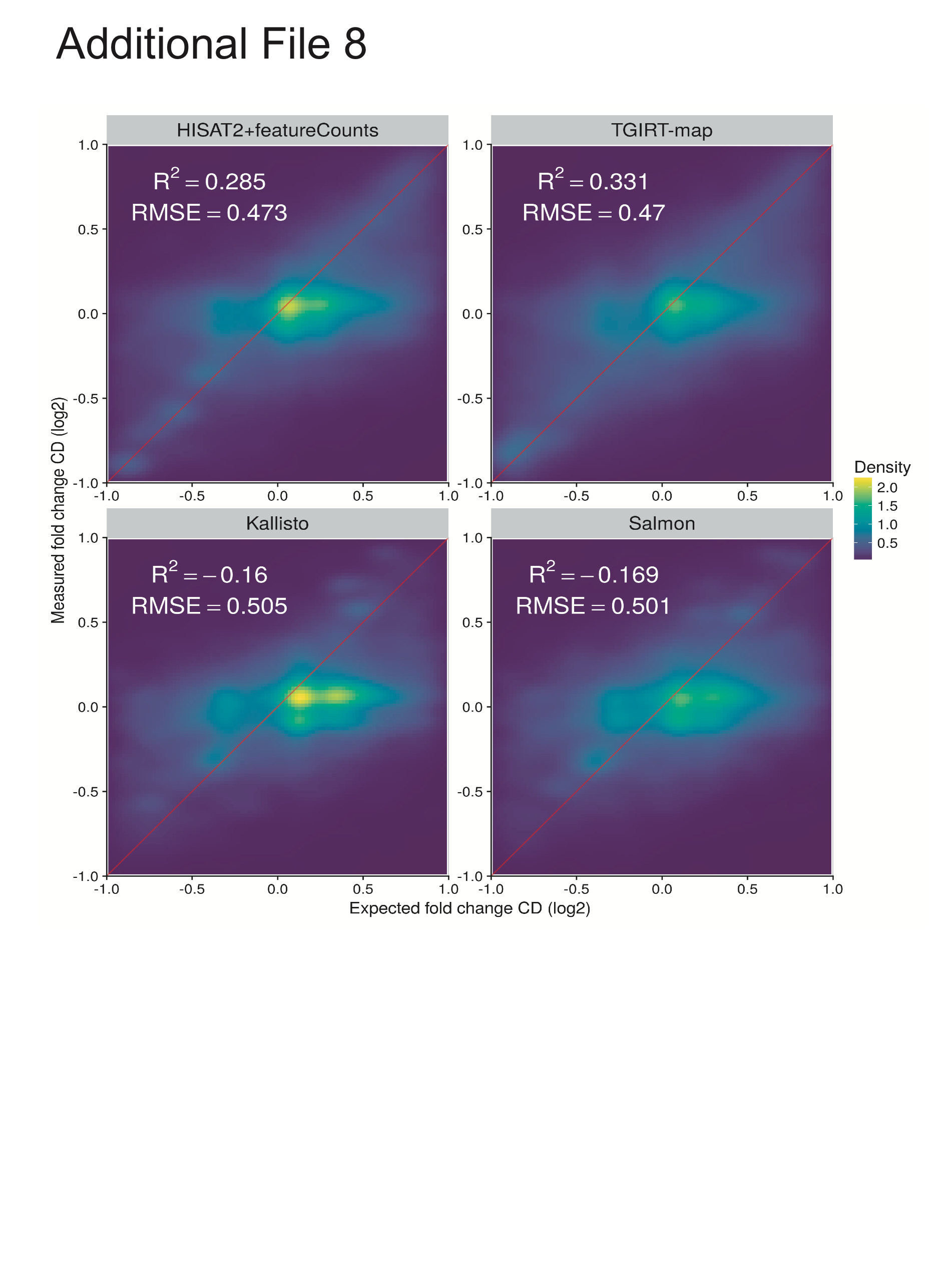

Supplement: Supplementary file 8 — Measured versus expected log2 fold-changes between samples C and D for the shortest 25% genes from each pipeline. Expected log2 fold-changes for each gene (denoted by red line) were constructed from (A) the measured log2 fold-changes between samples A and B, and (B) the known sample mix ratio of samples A and B in samples C and D, using a previously-described algorithm [19]. Each panel shows a two-dimensional kernel density estimation of the distribution of measured vs expected log2 fold-changes between samples C and D for genes in the lowest gene length quantile. Coefficients of determination for each group of genes are annotated as R2. Perfectly correlated log2 fold-changes are annotated by the diagonal red lines. (PNG 428 kb) [file 12864_2018_4869_MOESM8_ESM.png]

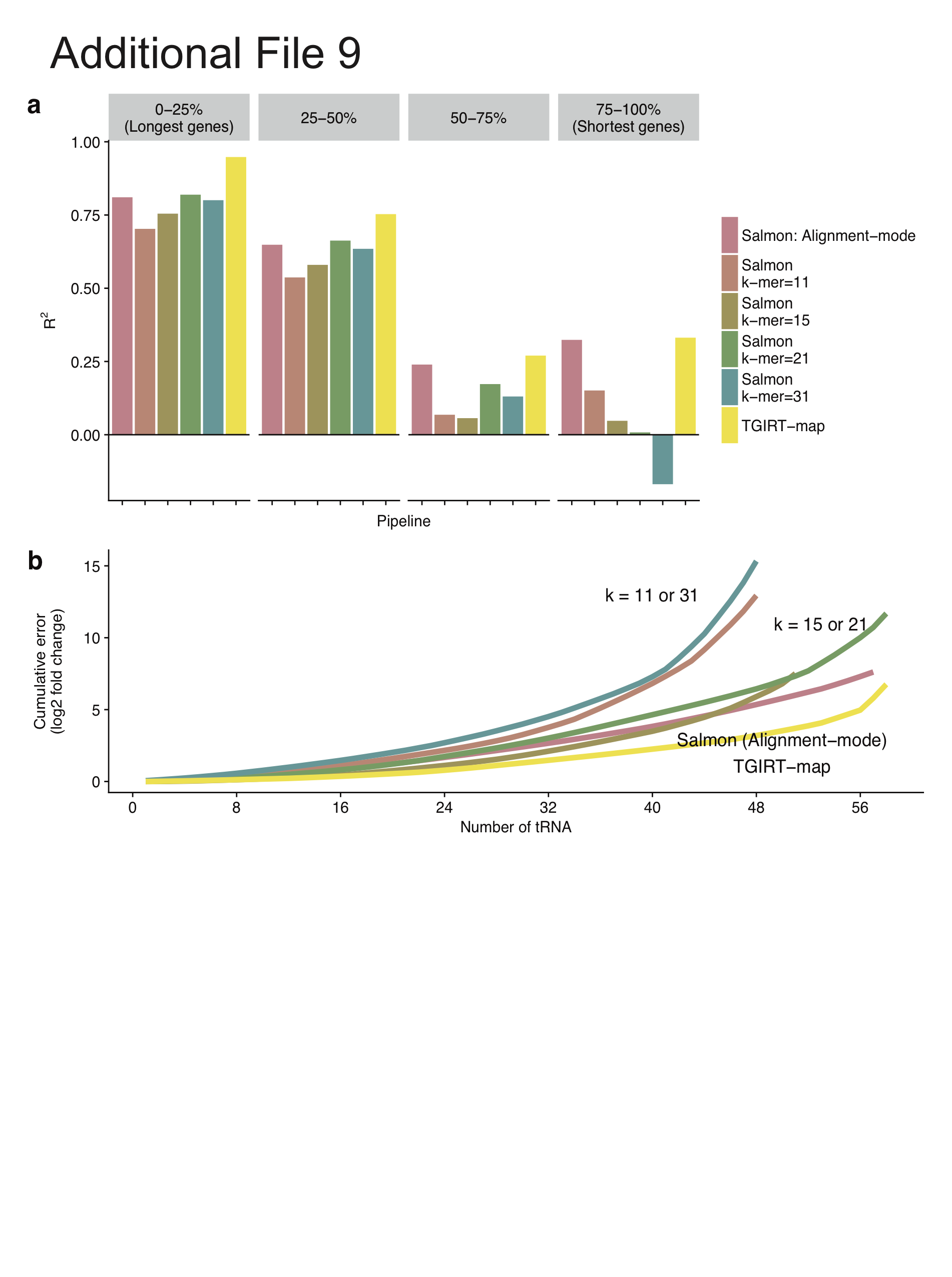

Supplement: Supplementary file 9 — Performances of Salmon using alignment-mode and different k-mer sizes of 11, 15, 21, and 31 (default). (a) Accuracies of fold-change estimation in quantile groups of gene lengths. All detected genes from TGIRT-map or Salmon with different k-mer sizes were grouped by their gene lengths into four quantile groups. For each quantile group, R2 values were computed from the expected and measured log2 fold-change values between samples C and D. Bars are color-coded by pipelines. Coefficients of determination (R2) were computed by R2 function from R caret package [35]. (b) Cumulative absolute errors in tRNA log2 fold-change predictions for each k-mer size for Salmon and TGIRT-map. tRNAs were sorted in ascending order by their absolute errors in log2 fold-change predictions. Cumulative absolute errors for all detected tRNA were plotted. Lines are color-coded by pipeline. (PNG 286 kb) [file 12864_2018_4869_MOESM9_ESM.png]

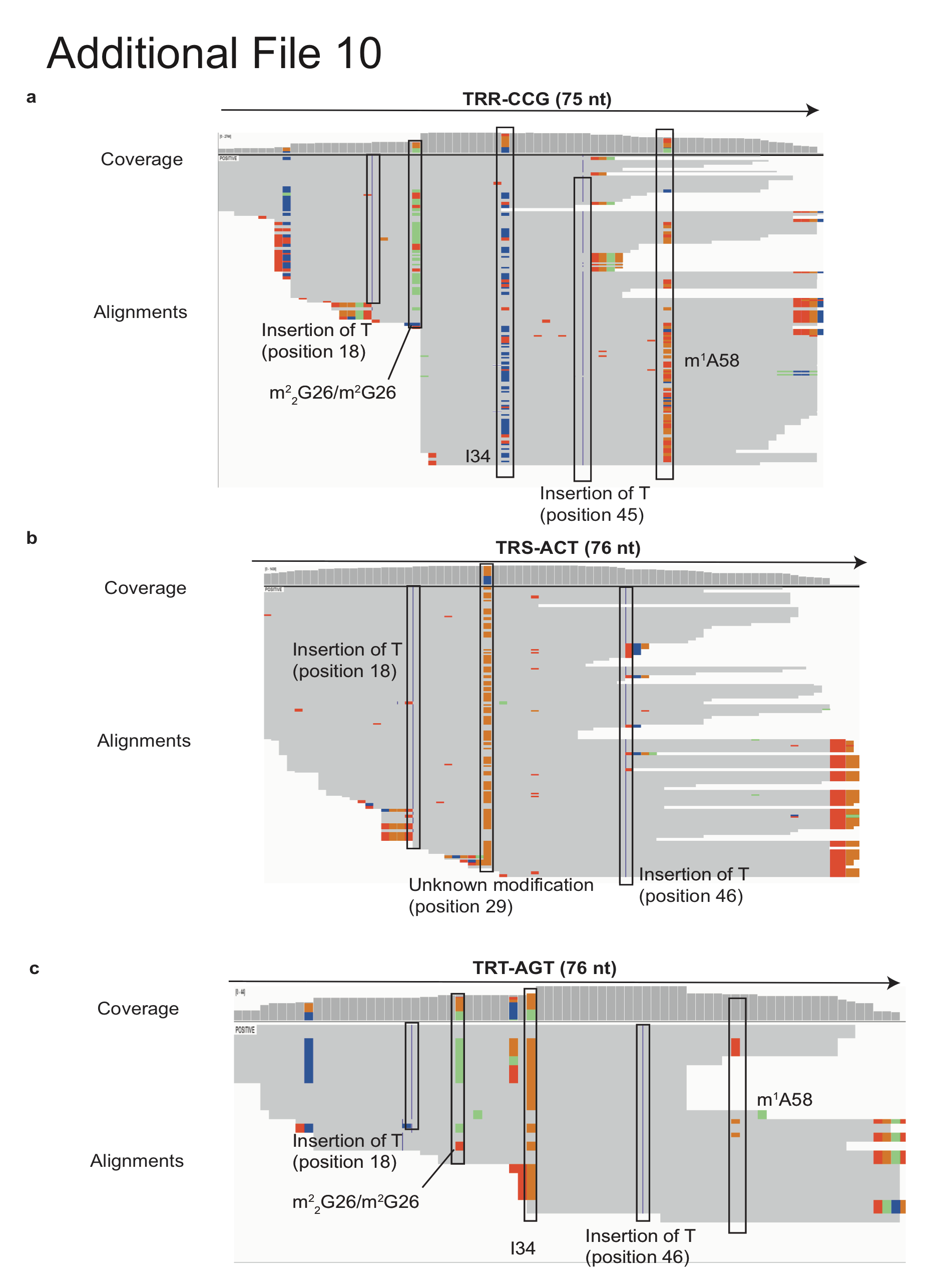

Supplement: Supplementary file 10 — Erroneous sequencing reads may have impeded k-mer counting for tRNAs. IGV genome browser screen-shots of three tRNA alignments from Sample A1 aligned via TGIRT-map were shown. The three tRNA alignments being shown are (a) TRR-CCG, (b) TRS-ACT, and (c) TRT-AGT. Arrows on the top of each panel indicate the corresponding tRNA transcript strands (5’ to 3’) and sizes. Bar charts below the arrows represent coverages at every position along the tRNA transcripts. Read alignments from Sample A1 aligned by TGIRT-map are shown below the bar charts. Grey colors represent read bases that match the reference bases. Other colors indicate bases on the read alignments that do not match the reference bases (purple, red, green, blue, and brown colors indicate insertions, thymidines, adenosines, cytidines, and guanosines on the read alignments, respectively). These errors may reflect post-transcriptionally-modified RNA bases, which interfere with canonical base-pairings and lead to misincorporations during TGIRT reverse transcriptions [15, 17, 26]. Errors due to misincorporations at known and unknown sites of post-transcriptionally modified RNA bases are highlighted with boxes (m$^{2}_{2}$22G26: N2,N2-dimethylguanosine at position 26;m2G26: N2-methylguanosine at position 26; I34: Inosine at position 34; m1A58: 1-methyladenosine at position 58; see also [15, 17, 36]). Unannotated mismatches from both ends can be untrimmed adapters or tRNA-precusor sequences. We observe that on these tRNAs, the errors induced by RNA-base-modification occurred at positions ∼ 20, 30, or 50 on ∼ 75 nucleotide long tRNA transcripts. Therefore, we expect that every k-mer originating from an erroneous sequencing read inherits at least one of these errors, and therefore k-m [file 12864_2018_4869_MOESM10_ESM.png]
